# Antioxidative and antiphotoaging activities of neferine upon UV-A irradiation in human dermal fibroblasts

**DOI:** 10.1042/BSR20181414

**Published:** 2018-12-14

**Authors:** Abidullah khan, Hongliang Bai, Maoguo Shu, Mingxia Chen, Amin Khan, Zhuanli Bai

**Affiliations:** 1 Department of Burn and Plastic Surgery, First Affiliated Hospital of Xi’an Jiaotong University, School of Medicine, 277 West Yanta Road, Xi’an, Shaanxi 710061, China; 2 Department of Electron Microscopy, Xi’an Jiaotong University, School of Medicine, 277 West Yanta Road, Xi’an, Shaanxi 710061, China; 3 Department of Chemistry, University of Science and Technology, Bannu, Khyber Pakhtunkhwa (KPK) 28100, Pakistan

**Keywords:** Antioxidant, Fibroblasts, Neferine, Oxidative stress, Photoaging, Ultraviolet A

## Abstract

Our daily exposure to ultraviolet radiation (UVR) results in the production of reactive oxygen species (ROS), lipids, proteins and DNA damage and alteration in fibroblast structure, thus contributing to skin photoaging. For this reason, the use of natural bioactive compounds with antioxidant activity could be a strategic tool to overcome ultraviolet A (UV-A) induced deleterious effect. Neferine is an alkaloid extract from the seed embryos of lotus (*Nelumbo nucifera* Gaertn). In the present study, we report the protective effect of neferine against UV-A induced oxidative stress and photoaging in human dermal fibroblasts (HDFs). HDFs subjected to UV-A irradiation showed increased production of ROS and malondialdehyde (MDA). Furthermore, it depleted the cellular enzymatic antioxidant superoxide dismutase (SOD) and non-enzymatic antioxidant glutathione peroxidase (GPx). On the other hand, HDFs treated with neferine followed by UV-A irradiation reversed the process, reduced the ROS and lipid peroxidation and restored the antioxidants pool. Moreover, neferine treatment significantly inhibited UV-A induced matrix metalloproteinase-1 (MMP-1) expression in HDFs. Remarkable morphological and ultrastructural alterations observed in HDFs upon UV-A irradiation, were also reduced with neferine treatment. Taken together, our results suggest that neferine has strong antioxidative and photoprotective properties and thus may be a potential agent for the prevention and treatment of UV-A mediated skin photoaging.

## Introduction

Chronic UV exposure is the primary cause of premature skin aging also called photoaging [1]. Photoaged skin is characterized by loss of skin tone and resilience, roughness and dryness, irregular pigmentation and deep wrinkles formation [2]. The principal mode of UV-A action is ROS generation [3]. ROS is not only damaging biological macromolecules (DNA, carbohydrates, lipids and proteins), but also capable of depleting antioxidants such as SOD and GPx [4,5]. The increasing ROS activity degrades the polyunsaturated fatty acids of the cell membrane causing membrane lipid peroxidation [6]. Lipid peroxidation is a crucial pathophysiological event in many diseases such as aging, cancer, diabetes, cardiovascular diseases and rheumatoid arthritis [7]. Malondialdehyde (MDA) is the enzyme biomarker and the end product of lipid peroxidation. This compound is a reactive aldehyde and is a marker to measure the oxidative stress in an organism [8].

Skin photoaging is also associated with the up-regulation of metalloproteinases (MMPs) in skin dermal fibroblasts upon UV exposure. MMPs have structurally related zinc-dependent endopeptidases that play a crucial role in the degradation of extracellular matrix (ECM) in connective tissues [9]. Among them, MMP-1 is the interstitial collagenase that initiates the degradation of type I and III fibrillar collagens [10]. In short, high production of ROS results in overexpression of MMP-1 causing collagen and elastin degradation, playing a crucial role in skin aging. Based on the underlying mechanism, it is essential to utilize certain agents that have antioxidative and antiphotoaging properties to attenuate skin photoaging.

Mitochondria are known to be the powerhouse of a cell. During the process of oxidative phosphorylation or ATP synthesis, misdirection of an electron leads to the generation of ROS which makes mitochondria the highest ROS turnover in cell [11]. Increased oxidative stress is linked with degradation of the endoplasmic reticulum (ER) and Golgi apparatus (GA), but the molecular process is still far from being understood. Since oxidative stress has been associated with the pathogenesis of various diseases, we explored the possibility of high ROS expression upon UV-A exposure being associated with degeneration and degradation of ER and GA.

In the past few decades, we have seen the rising interest in the use of plants and their derivatives for the prevention and treatment of UV induced photoaging. Natural compounds are traditionally used in the treatment of skin rejuvenating and photoprotective cosmetic formulations [12]. Recently, strawberry based formulations have been proposed for the prevention of UV-A exposure-induced oxidative damage in HDFs [13,14]. Aaptamine is an alkaloid isolate from the marine sponge which has been reported to have antioxidant and antiphotoaging properties [15].

**Figure 1:**
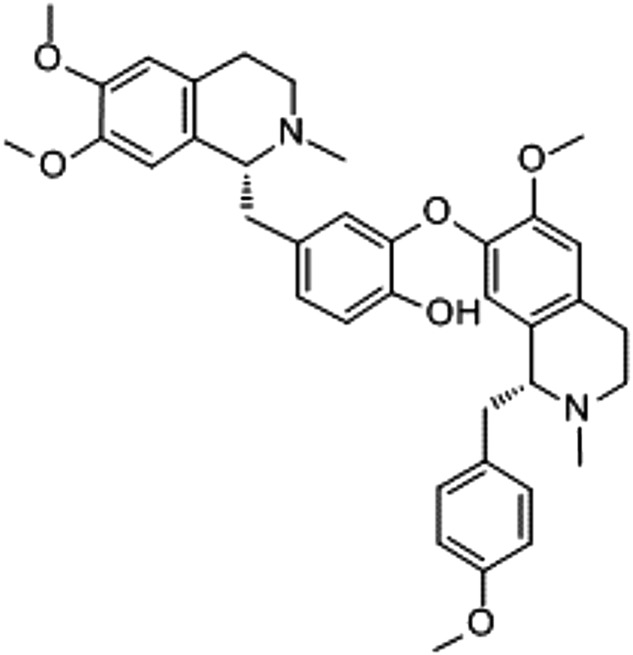
Structure of neferine The empirical formula of neferine is C_38_H_44_N_2O6_, with a relative molecular weight of 624.

Many skin care products use lotus plant extract as an antiaging compound. The antioxidant activities of the lotus plant are well established, e.g. leaf [16], stamens [17] and rhizomes [18]. Lotus seed embryos are commonly used in traditional Chinese medicine and consumed as tea ingredient or eaten raw which is believed to have antiaging properties. Neferine is one of the major bisbenzylisoquinoline extract derived from seed embryos of lotus (Figure 1) along with liensinine and isoliensinine [19]. The various pharmacological activities of neferine have been reported including the anti-arrhythmia and anti-hypertensive [20], antidiabetic [21] and sedative properties [22]. Some studies have shown the antioxidant and anti-inflammatory properties of neferine [23]. However, its protective and therapeutic effect on the prevention of skin photoaging has never been proposed.

Consistent with the reports mentioned above, in the present study, we wanted to analyze the antioxidant and antiphotoaging properties of neferine by creating an *in vitro* model of human dermal fibroblasts followed by UV-A irradiation. We aimed to investigate whether neferine could attenuate oxidative stress by testing ROS and MDA levels and oxidative indicators such as SOD and GPx activities. We also determined the matrix-degrading enzyme, MMP-1 expression in UV-A irradiated HDFs pretreated with neferine. Furthermore, UV-A induced morphological and ultrastructural alterations in HDFs were studied to evaluate the cytoprotective properties of neferine.

## Materials and methods

### Cell culture and neferine treatment

**Figure 2:**
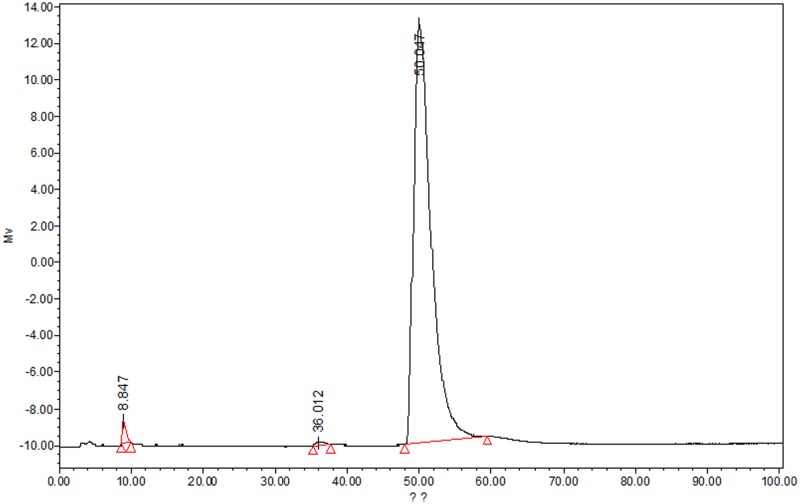
Chromatogram of neferine extract from lotus seeds at 280 nm

The HDFs (lot no. 61447289), obtained from American Type Culture Collection (ATCC, Manassas, VA, U.S.A.) was a gift from Dr. Zhao Ban (Department of Burn and Cutaneous Surgery, Xijing Hospital, Xi’an, Shaanxi, China). Cells were cultured in Dulbecco’s modified eagles medium (DMEM) containing 10% heat-inactivated fetal bovine serum (FBS) and 1% 100 IU/ml penicillin and 100 µg/ml of streptomycin at 37°C in a humidified incubator with 5% CO_2_. Cells confluence reaching 80-90% were digested with 0.25% trypsin and passaged. The cells used in the present study were in a logarithmic growth phase and passaged between 3 to 12. Cultured cells were in a serum-free medium for 24 h before when they were subjected to any experimentation. Subconfluent cultures were washed twice with phosphate buffered saline (PBS) and treated with various concentration of neferine (Pure One Biotechnology Laboratories, Shanghai, China) 1 h before UV-A irradiation. The purity of neferine was found to be ≥98.0% using HPLC method (Figure 2). HDFs were treated for 40 min with a total of 20 J/cm^2^ UV-A dose. Philips TL-K (200233 Shanghai, China) was used as a source of UV-A. Control group HDFs were treated the same except for UV-A irradiation. Following irradiation, cells were incubated for 24 h (with or without neferine) and harvested for further experimentation.

### Cell viability assay

The cytoprotective effect of neferine was determined by MTT assay (Nanjing Jiancheng Bioengineering Institute, Nanjing, China) kit. HDFs were seeded in a 96-well plate at a density of 1 × 10^4^ cells/ well. After 24 h of incubation, the cells were treated with a medium containing various concentrations of neferine (0.025, 0.05, 0.1, 0.2, 0.4, 0.8, 1.6, 3.2, 6.4 and 12.8 µM). The following day 50 µl (5 mg/ ml PBS) of MTT assay was added to each well. Following 4 h of incubation, the media was removed and 150 µl of dimethylsulfoxide (DMSO) was added to solubilize the formazan crystals formed. The absorbance of samples was measured at 492 nm using a spectrophotometer (Multi-Skan, MK3, Thermo Electron, Waltham, MA, U.S.A.). Each assay was repeated in triplicate, and the mean was calculated.

### Light and transmission electron microscopy

Morphological changes in HDFs following UV-A irradiation were observed using a light microscope (Nikon Eclipse Ti-S) and photographed (Nikon Digital Sight DS-U3). Passage 4 HDFs were cultured in 100 mm dishes, and serum was removed 24 h before its treatment with neferine. Cells were treated with or without neferine 1 h before UV-A irradiation. Following 24 h of incubation, the cells were washed with PBS and observed under the microscope. For ultrastructural analysis, HDFs were first fixed in 2.5% glutaraldehyde and 4% paraformaldehyde in phosphate buffer for 2 h at 4°C and then with 0.1 M phosphate buffer for 30 min. HDFs were treated with 1% osmium tetroxide in phosphate buffer for 2 h at 4°C followed by rinsing with 0.1 M PBS two to three times. Cells were dehydrated with graded ethanol 30%, 50%, 70%, 90%, 100% and propylene oxide for 10 min. The cells were immersed in a mixture of propylene oxide and Epon 812 for 1 h at 60°C and then embedded at 60°C for 48 h. A 50 nm thin sections (LKB Ultratome, Sweden) were stained with Azur II Toluidine blue guided by a light microscope. Sections were then stained with uranyl acetate and lead hydroxide for 10 min and observed under an electron microscope (Hitachi H-7650, Japan).

### Detection of ROS and MDA activities

The intracellular ROS levels were measured using 2,7-diacetyl dichlorofluorescein diacetate (DCF-DA) method according to ROS (cat no: E004, Nanjing Jiancheng Bioengineering Institute, Nanjing, China) assay protocols. HDFs were incubated with various concentrations of neferine 1 h before UV-A irradiation. Following 24 h of incubation, they were washed twice with PBS and incubated with DCF-DA (1:500) for 30 min. Fluorescence was determined using a microplate fluorospectrophotometer (2030 Multilabel Reader Victor X5, PerkinElmer, Waltham, MA, U.S.A.). The fluorescence had a maximum wave crest of 502 nm excitation wavelength and 530 nm for emission wavelength. The fluorescence intensity is proportional to the level of intracellular ROS.

For measuring MDA levels (cat no: A003-2, Nanjing Jiancheng Bioengineering Institute, Nanjing, China), HDFs were collected and centrifuged at 1000 rpm for 10 min. The supernatant was discarded and cells pellet was resuspended in 1 ml of PBS followed by centrifugation at 1000 rpm for 10 min. The cells pellet was homogenized with 0.4 ml of physiological saline. MDA, which is lipid peroxidation of polyunsaturated fatty acid induced by free radicals within cell condensate with thiobarbituric acid (TBA) was measured spectrophotometrically using absorbance at 532 nm.

### Assay of antioxidants levels

Intracellular SOD and GPx activities were measured using SOD (cat no: WST-1, A001-3) and GSH-GPx (cat no: A005), (Nanjing jiangcheng Bioengineering Institute, Nanjing, China) assay kits. HDFs seeded in 100 mm culture dishes were treated with or without neferine for 1 h before they were subjected to UV-A irradiation. Following 24 h of incubation, HDFs were trypsinized and centrifuged at 1000 rpm for 10 min. The supernatant was discarded and the sediment HDFs were resuspended in 1 ml of PBS and centrifuged at 1000 rpm for 10 min. HDFs were homogenized using sonication (Sonics Vibra Cell, Newtown, CT 06470, U.S.A.). SOD and GPx assays were measured spectrophotometrically (Multiskan, Thermo Scientific, Waltham, MA, U.S.A.) using absorbance levels of 512 and 405 nm respectively.

### Western blotting

HDFs pretreated with various concentrations of neferine followed by UV-A irradiation were collected and rinsed with PBS. They were then lysed with 0.2 ml of radioimmuno precipitation assay (RIPA) buffer (HEART biological technology, China) on ice. Lysates were collected and centrifuged at 10000 rpm for 10 min at 4°C. 20 µl supernatant was used to quantify total protein concentration. The lysate was denatured by mixing it with 50 µl of protein loading buffer at 100°C for 5 min. The calculated amount of protein samples was loaded on a 10% sodium dodecyl sulfate (SDS)-polyacrylamide page for electrophoresis and then transferred to a polyvinylidene fluoride (PVDF) membrane. Western blot was performed for analysis of MMP-1 expression using specific antibodies. The band signals were visualized on LAS-400 Lumino Image Analyzer (Fujifilm, Tokyo, Japan) using a Millipore detection reagent at a ratio of 1:1 (Millipore, Billerica, MA, U.S.A.) the relative amount of proteins associated with specific antibodies, anti-MMP1 antibody (cat no: ab38929, Abcam) and B-actin (cat no: SC-47778, Santa) were normalized according to the intensities of B-actin. The band intensities were quantified using MultiGauge software (Bio-Rad, Hercules, CA, U.S.A.) image analyzer.

### Assay of protein concentration

Bicinchoninic acid (BCA) (cat no: A-045-3, Nanjing Jiangcheng Bioengineering Institute, Nanjing, China) method was used to measure the protein content in fibroblasts. Briefly, cells were trypsinized and centrifuged at 1000 rpm for 10 min. The supernatant was removed and HDFs were homogenized in PBS. Homogenate was incubated for 30 min at 37°C and absorbance was measured at 560 nm.

### Statistical analysis

The statistical analysis was performed using GraphPad Prism 5 (GraphPad Software, La Jolla, CA). Data analysis was carried out by the variance of one-way ANOVA followed by the Bonferroni multiple comparison tests. *P*<0.05 was considered statistically significant. Collected data was presented as the mean ± SD of (*n*=4) independent experiments.

## Results

### Cytoprotective effect of neferine

**Figure 3:**
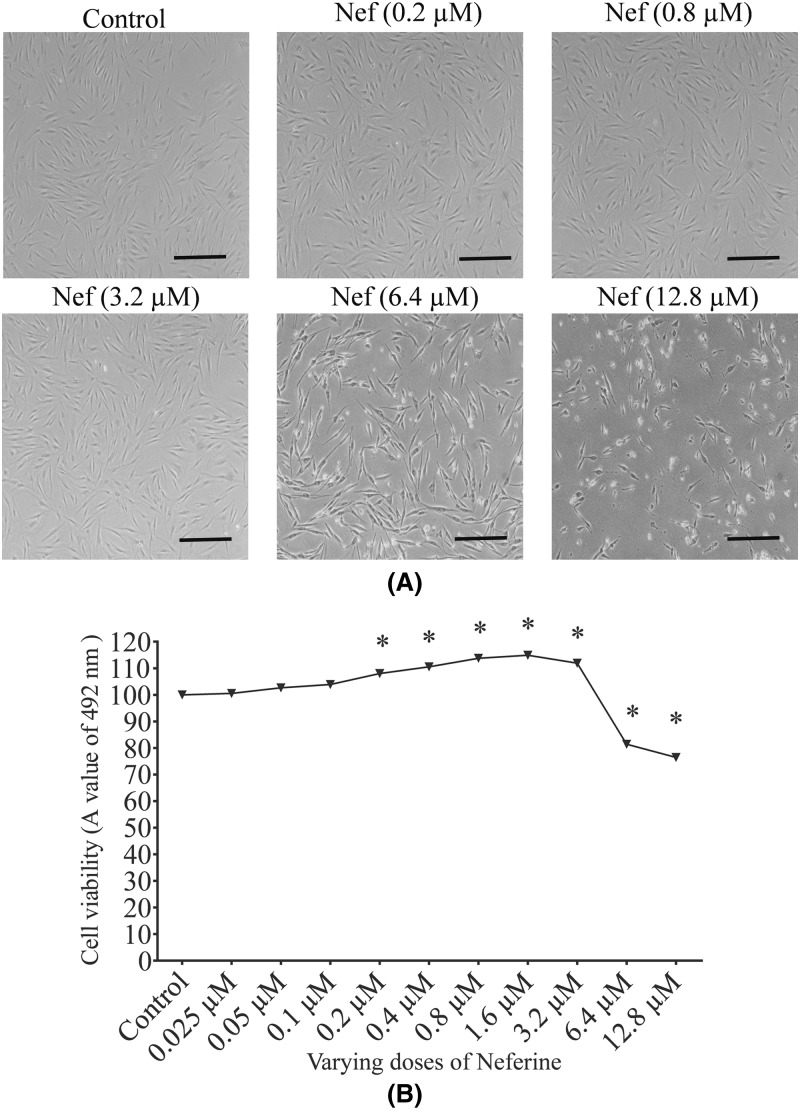
Cytoprotective effect of neferine (Nef) in human dermal fibroblasts (**A**) Effect of neferine on the morphology of fibroblasts. Original magnification 100x; scale bar 100 µm. (**B**) protective effect of neferine was analyzed using the MTT assay. Data represents the ± SD mean (*n*=4). The significance of difference versus the control group is *P<0.05.

The protective nature of neferine was analyzed by incubating HDFs with various concentrations of neferine (0.025, 0.05, 0.1, 0.2, 0.4, 0.8, 1.6, 3.6, 6.4, and 12.8 µM) for 24 h. The cell viability of HDFs increased in a concentration-dependent manner. Significant reduction in HDFs growth was observed with neferine concentration 6.4 and 12.8 µM, indicating the toxicity of neferine at higher doses (Figure 3B). When HDFs were observed under a light microscope, they displayed no marked morphological changes till 3.2 µM concentration of neferine. The cells at 6.4 µM of neferine concentration, showed sharp or thinner extension and lost their intercellular connection compared with the control group. The cells treated with a neferine concentration 12.8 µM were rounded and shrunken and were detached from the culture dish (Figure 3A). These results indicate that neferine at lower concentration has proliferative properties but at higher concentration can cause cell apoptosis and death. There is a constant increase in cell viability at 0.2, 0.4, and 0.8 µM concentrations of neferine. Based on previous studies [24] and our observations, 0.2, 0.4 and 0.8 µM of neferine were considered to be suitable for experimentation.

### Neferine prevents morphological and ultrastructural alterations in UV-A irradiated HDFs

**Figure 4:**
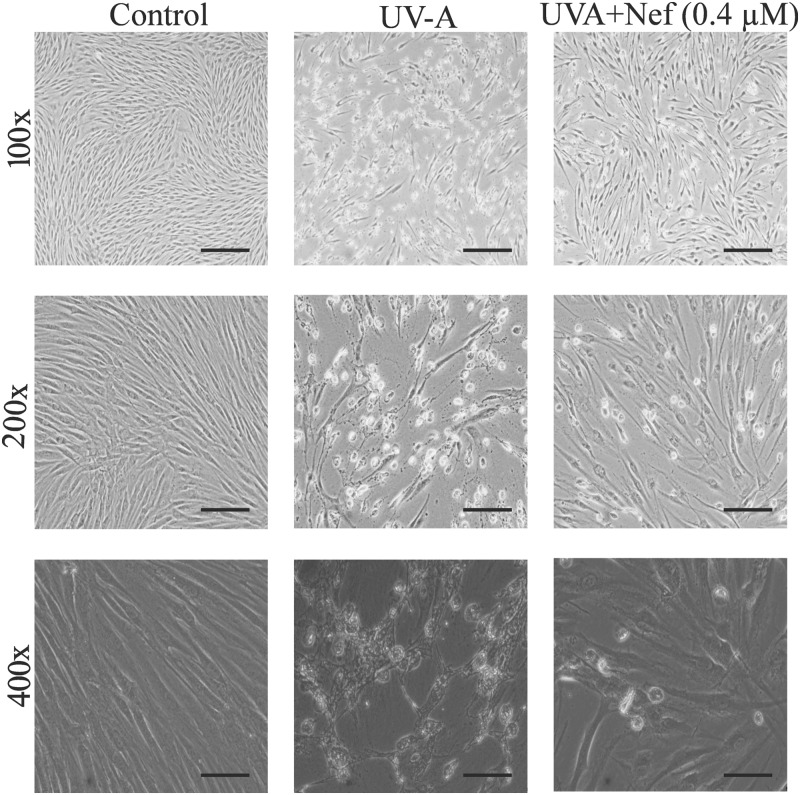
Protective role of neferine (Nef) in human dermal fibroblasts following UV-A irradiation Magnification 100x; scale bar 100 µm, 200x; scale bar 100 µm and 400x; scale bar 50 µm.

HDFs pretreated with or without neferine followed by UV-A irradiation were collected and assayed for morphological and ultrastructural changes. Control group HDFs showed centrally located nuclei with radiating flame or whirlpool shapes (Figure 4). However, HDFs treated with UV-A alone showed signs of a loosened intercellular connection, rounded body and blebbing were apparent features. The UV-A alone treated group displayed inhibited cell proliferation, widened cell interval and were detached from the cell culture dish (Figure 4). On the other hand, UV-A+Nef (0.4 µM) treated group displayed reduced morphological changes and less apoptotic cell bodies compared with UV-A alone group (Figure 4).

**Figure 5:**
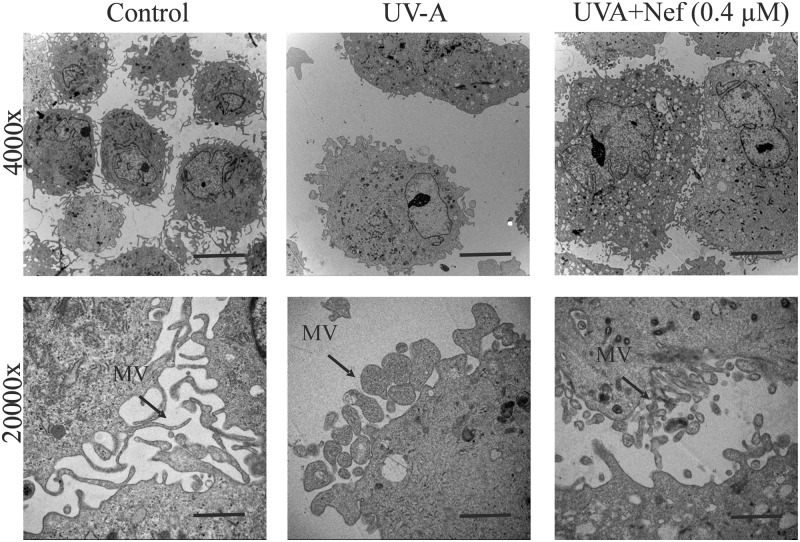
TEM showing the protective effect of neferine (Nef) in HDFs upon UV-A irradiation Magnification 4000x; scale bar 5 µm and 20000x; scale bar 1 µm.

**Figure 6:**
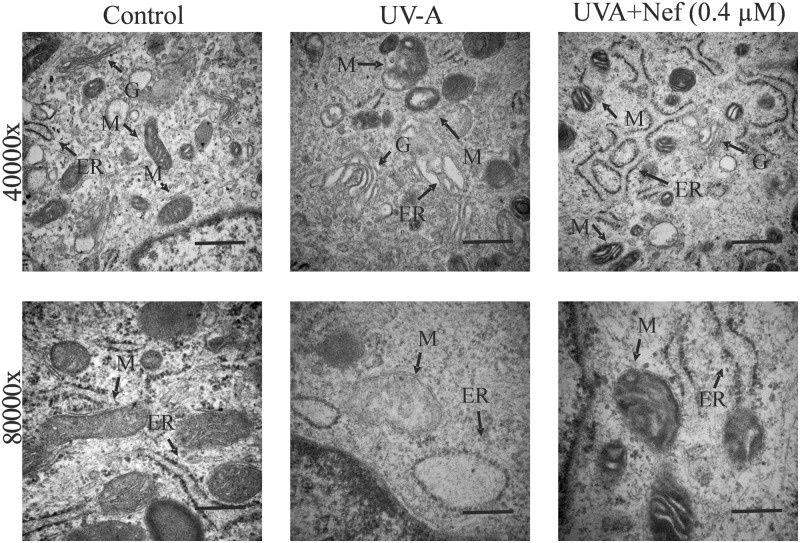
TEM showing the protective role of neferine (Nef) on mitochondria, ER, and GA in human dermal fibroblasts following UV-A irradiation Magnification 40000x; scale bar 500 µm and 80000x; scale bar 200 µm.

Ultrastructural analysis revealed microvilli shading and blunting in UV-A alone treated HDFs compared with the control group, where the HDFs had long and thin microvilli (Figure 5). The HDFs in UV-A+Nef (0.4 µM) group had fewer microvilli shading and blunting (Figure 5). Control group displayed normal ER, GA and mitochondrial architecture (Figure 6). Mitochondrial swelling and loss of cisternae were apparent in the UV-A alone group. UV-A irradiation caused ER and GA fragmentation and swelling in HDFs. On the other hand, UV-A+Nef (0.4 µM) treated group showed nearly normal structures, i.e. lesser mitochondrial deformity and reduced ER and GA swelling and fragmentation. (Figure 6).

### Neferine reduced intracellular ROS activity in HDFs

**Figure 7:**
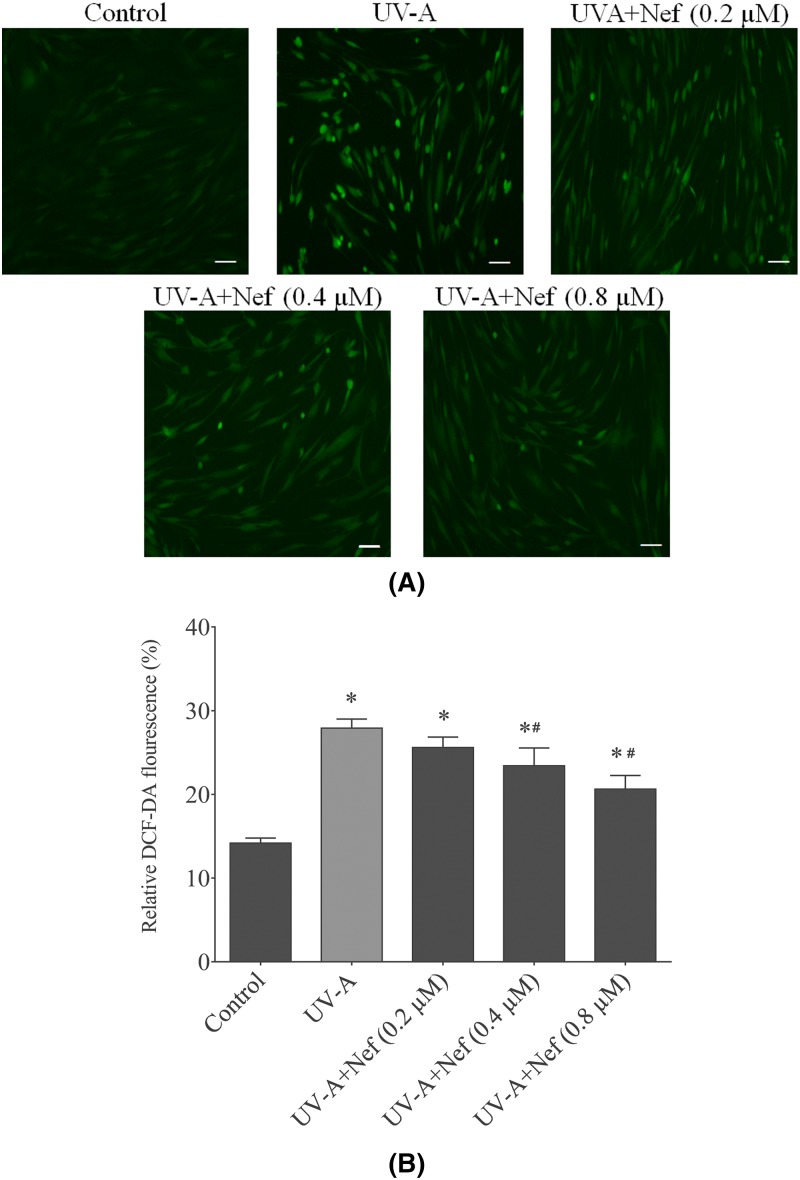
Effect of neferine (Nef) on ROS generation in human dermal fibroblasts upon UV-A irradiation (**A**) DCF-DA fluorescence is an indication of the relative intensity of ROS production in mitochondria. (**B**) The 499 and 525 nm were the excitation and emission wavelengths respectively. Data shown are the representation in ±SD (*n*=4). *P<0.05 versus control group; ^#^*P*<0.05 versus UV-A alone group.

The impact of neferine on intracellular ROS relative fluorescence intensity in HDFs was examined following UV-A irradiation (Figure 7A). HDFs in the UV-A alone treated group showed a significant increase in ROS activity compared with the control (*P<0.05) and the neferine treated group (^#^*P*<0.05). Neferine application significantly inhibited intracellular ROS generation in a concentration-dependent manner (Figure 7B).

### Neferine reduced MDA level in HDFs

**Figure 8:**
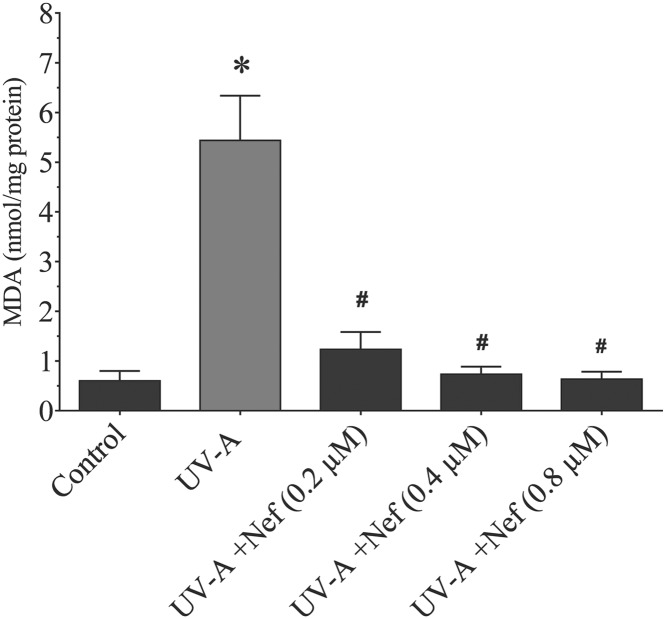
Effect of neferine (Nef) on MDA levels in human dermal fibroblast upon UV-A irradiation Data are the representation in ±SD (*n*=4). *P<0.05 versus control group and ^#^*P*<0.05 versus UV-A alone group.

MDA is a biomarker of lipid peroxidation, which was assayed as TBA and measured as reacting substance thiobarbituric acid reactive substances (TBARS). There was a significant increase in MDA activity in UV-A alone group compared with the control group (*P<0.05). On the other hand, dose-dependent reduction in MDA levels were noticed in the neferine treated groups compared with the UV-A alone group (^#^*P*<0.05) and control group (*P<0.05) (Figure 8).

### Antioxidant effect of neferine

**Figure 9:**
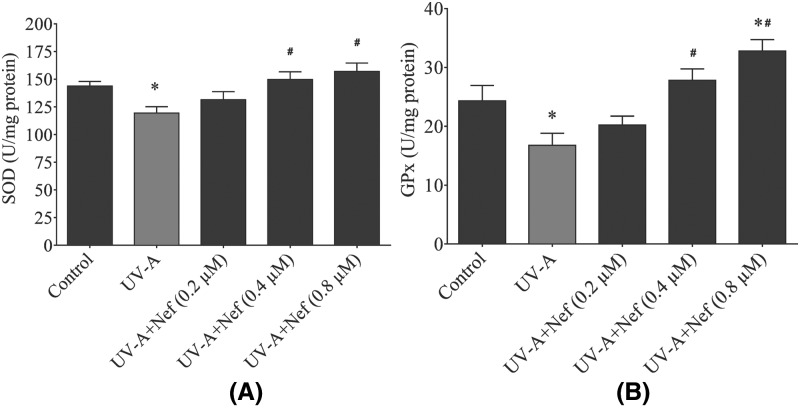
Effect of neferine (Nef) on (**A**) SOD and (**B**) GPx activities in human dermal fibroblasts following UV-A irradiation Data are expressed as the mean ± SD of *n*=4.*P<0.05 versus control group and ^#^*P*<0.05 versus UV-A alone group.

HDFs were treated with neferine for 1 h and then exposed to UV-A irradiation. Following 24 h of incubation, SOD, GPx levels were assayed. The SOD activity was significantly reduced in the UV-A alone group compared with the control group (*P<0.05). The SOD activity was increased in a concentration-dependent manner in the neferine-treated groups compared with the control group (*P<0.05) and the UV-A alone group (^#^*P*<0.05), (Figure 9A). The non-enzymatic activity of GPx was significantly reduced in UV-A alone group compared with control group (*P<0.05) and the neferine-treated groups (^#^*P*<0.05). HDFs in neferine-treated group increased the GPx activity in a concentration-dependent manner (Figure 9B)

### Neferine reduced the MMP-1 expression in HDFs

**Figure 10:**
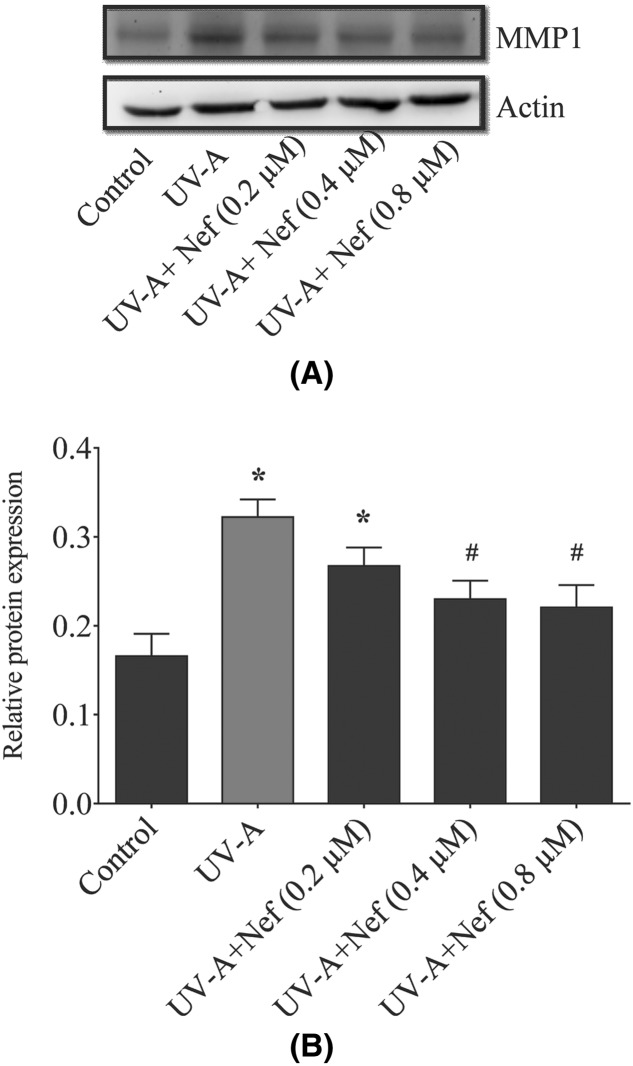
Inhibitory effect of neferine (Nef) on MMP-1 expression in human dermal fibroblasts upon UV-A irradiation (**A**) Western blotting analysis of MMP-1 expression in HDFs. (**B**) The data are expressed as the mean ± SD of *n*=3. *P<0.05 versus control group and ^#^*P*<0.05 versus UV-A alone group.

Fibroblasts were treated with various concentrations of neferine followed by UV-A irradiation. UV-A alone group showed increased expression of MMP-1 compared with the control group (*P<0.05). On the other hand, HDFs treated with neferine significantly reduced MMP-1 expression in a concentration-dependent manner compared with the UV-A alone group (^#^*P*<0.05) and control group (*P<0.05) (Figure 10A,B).

## Discussion

One of the best strategies to minimize UVR harmful impact on the skin is to replenish the antioxidant pool and to suppress collagen degradation using natural phytochemicals. Plants and their extracts are widely used in cosmetics because they are relatively harmless and possess multiple beneficial properties. Studies have shown the antioxidant and antiaging properties of natural compounds [25]. Plant extracts contain bioactive substances such as isoflavones, anthocyanins and catechins may have antioxidant activity [26]. Polyphenols isolated from grape seed, green tea, strawberry anthocyanins, luteolin, silymarin and genistein have shown to prevent cellular photodamage [27,28].

Lotus seed embryos have been used in traditional Chinese medicine for centuries and it is believed to possess antiaging properties. The antioxidant and anti-inflammatory properties of neferine have been studied previously [23,29]. Therefore, exploring its therapeutic effect on prevention of skin photoaging will be very useful. In this present study, we demonstrated the antioxidative and antiphotoaging properties of neferine in HDFs upon UV-A irradiation. UV-A induced ROS generation plays a crucial role in photoaging, immune suppression, and photocarcinogenesis by influencing dermal fibroblasts [30]. Neferine can specifically decrease the ROS levels [31]. we found that neferine reduced the ROS generation in HDFs and protected the cells from free radical-induced cellular damage. All cells have antioxidant defence system to compensate and neutralize the effect of ROS and lipid peroxidation. Among naturally occurring antioxidants SOD, GPx and catalase (CAT) play an essential role. SOD is one of the most crucial enzymatic antioxidants. SOD converts superoxide anions into H_2_O_2_ and O_2_, which is substantially less toxic than superoxide. SOD viability is the indirect measure of ROS scavenging and dynamic balance in the body. Glutathione (GSH) is the primary intracellular non-enzymatic antioxidant defense against ROS and plays a central role in cellular oxidative stress reduction. GPx uses GSH as a hydrogen donor to reduce H_2_O_2_ and hydroperoxides into H_2_O and O_2_. The imbalance between the antioxidants and free radicals causes cell damage and inevitably cell death, which is either caused by a decrease in antioxidants or an increase in ROS [32]. In the present study, we found the SOD and GPx activity levels were significantly low following UV-A irradiation and it is consistent with a decrease in HDFs viability and confluence. Neferine treated HDFs showed significant increase in antioxidants, subsequently reducing oxidative stress and cellular damage. The antioxidant activity of neferine could be associated with the presence of the hydroxyl group in its structure [33].

MDA level is an important marker of lipid peroxidation [34] and it is elevated when the oxidative cellular defence is deficient. UV irradiation of human skin causes immediate oxidative damage of lipids and proteins [35] and it occurs most likely in the superficial skin because it is directly exposed to environmental stimuli [36]. Studies have shown that membrane lipid peroxidation aggravates with ROS production [37] and continuous UV exposure increase MDA activity in mice skin [38]. In our study, we found that MDA levels were reduced in the neferine treated UV-A groups. Lipid peroxidation inhibition could protect the HDFs cell membrane and consequently prevent dermal changes linked with the formation of wrinkles.

Studies have shown the role of MMPs in the process of photoaging and aging [39]. UV is known to induce the expression of MMP-1 in the mice epidermis *in vivo* [39]. Our results demonstrate that neferine treatment reduced the expression of MMP-1 in UV-A subjected HDFs. As the generation of ROS by UV irradiation [40] is considered to play a significant role in the activation of AP-1, which is the transcription factor stimulating the transcription of MMP genes [41]. In our study, neferine significantly increased the antioxidants and reduced the ROS level, thus reducing MMP-1 expression in HDFs. In this consideration, we hypothesize that the antioxidative effects of neferine may be one reasonable explanation for its antiphotoaging capability.

Mitochondrial cristae play a pivotal role in apoptosis in addition to its well-known power supply function [42]. ROS generation in mitochondria is related with electron transport chain, which makes it the highest point for oxidative stress and lipid peroxidation. Reduced SOD and GPx activities weaken the cells ability to eliminate oxidative molecules formed in mitochondria. In our study, we found that the HDFs ultrastructural changes, such as shedding and blunting of microvilli and mitochondrial deformity was reduced in the neferine-treated group which may occur in ROS-dependent mitochondrial signaling pathway. The neferine-treated groups exhibited reduced ROS levels in a concentration-dependent manner explains the correlation between ROS formation and mitochondrial degeneration. Increase in ROS and reduction in SOD and GPx activities observed after UV-A irradiation could further aggravates mitochondrial dysfunction. It is reported that UV irradiation inhibits protein synthesis in rat fibroblasts [43]. UV irradiation produces structural changes in diverse nucleic acids and proteins via ROS production [44]. UV-A irradiation leads to ER fragmentation and swelling may relate to high ER stress induced by ROS accumulation. In our study, neferine treated HDFs displayed less marked ER alterations, which could be associated with a decrease in UV-A induced oxidative stress. The GA is responsible for sorting, packaging, and modification of protein synthesized in the rough ER. Fragmentation and degeneration of Golgi cisternae has been observed in various types of cells upon irradiation [45], and GA morphology recovers in the presence of antioxidants [46]. In the present study, neferine treated HDFs showed lesser degradation and swelling of GA, which may relate to an increase in intracellular antioxidants and reduction in UV-A mediated oxidative stress.

## Conclusion

The use of natural UV protection formulations offer an effective strategy to prevent the damaging effects of UV. In this work, for the first time, we proposed the antioxidative and photoprotective properties of neferine. We believe that neferine can be used in therapeutic and cosmetic products against UV-A induced oxidative stress and skin photodamage. Although the regulatory pathway of neferine action remains to be elucidated, further research is encouraged to investigate the molecular and anti-inflammatory effect of neferine.

## Abbreviations

BCA: Bicinchoninic acid
DCF-DA: 2,7-diacetyl dichlorofluorescein diacetate
DMSO: dimethylsulfoxide
ER: endoplasmic reticulum
GA: Golgi apparatus
GPx: glutathione peroxidase
GSH: glutathione
HDFs: human dermal fibroblasts
MDA: malondialdehyde
MMP-1: matrix metalloproteinase-1
PBS: phosphate buffered saline
ROS: reactive oxygen species
SOD: superoxide dismutase
TBA: thiobarbituric acid
UV-A: ultraviolet A
UVR: ultraviolet radiation
