# Expression of Concern: Antioxidative and anti-photoaging activities of neferine upon UVA irradiation in human dermal fibroblasts

**DOI:** 10.1042/BSR-20181414_EOC

**Published:** 2021-02-17

**Keywords:** Antioxidant, Fibroblasts, Neferine, Oxidative stress, Photoaging, Ultraviolet A

The Editorial Office has been made aware of potential issues surrounding the scientific validity of this paper, hence has issued an expression of concern to notify readers whilst the Editorial Office investigates.

