# Correction: Antioxidative and anti-photoaging activities of neferine upon UV-A irradiation in human dermal fibroblasts

**DOI:** 10.1042/BSR-2018-1414_COR

**Published:** 2021-07-01

**Keywords:** Antioxidant, Fibroblasts, Neferine, Oxidative stress, Ultraviolet A

This Correction follows an Expression of Concern relating to this article previously published by Portland Press.

The Authors of the original article “Antioxidative and antiphotoaging activities of neferine upon UV-A irradiation in human dermal fibroblasts” (*Biosci Rep* (2018) **38**(6): BSR20181414; DOI: 10.1042/BSR20181414) would like to correct Figure 3A, which had been identified as containing an overlap in two of the panels (Nef (0.2μM) and Nef (0.8μM)).

**Figure 3:**
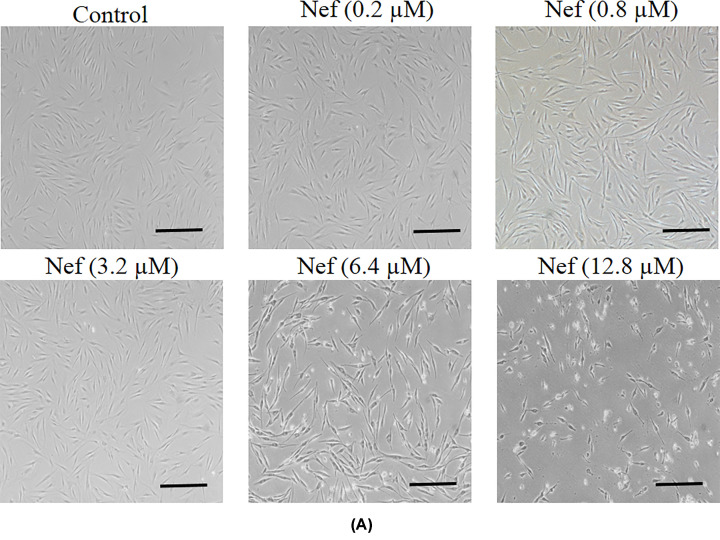
Cytoprotective effect of neferine (Nef) in human dermal fibroblasts (**A**) Effect of neferine on the morphology of fibroblasts. Original magnification 100x; scale bar 100 µm.

The Authors state that this error had occurred during the figure making process, and confirm that it does not affect the conclusions of their study. The correct version of Figure 3A is presented in this Correction.

